# Mapping gender and geographic diversity in artificial intelligence research: Editor representation in leading computer science journals

**DOI:** 10.1177/20584601231213740

**Published:** 2023-11-28

**Authors:** Felix Busch, Sarah Keller, Christopher Rueger, Avan Kader, Katharina Ziegeler, Keno K Bressem, Lisa C Adams

**Affiliations:** 1 Department of Radiology, 14903 Charité – Universitätsmedizin Berlin, Corporate Member of Freie Universität Berlin and Humboldt Universität zu Berlin, Berlin, Germany; 2 Division of Operative Intensive Care Medicine, Department of Anesthesiology, 14903 Charité – Universitätsmedizin Berlin, Corporate Member of Freie Universität Berlin and Humboldt Universität zu Berlin, Berlin, Germany; 3 Department of Radiology, Klinikum rechts der Isar, Technische Universität München (TUM), Munich, Germany; 4 Berlin Institute of Health at Charité – Universitätsmedizin Berlin, Berlin, Germany

**Keywords:** artificial intelligence, radiology, gender equity, bioethics, computer science, editorial policies

## Abstract

**Background:**

The growing role of artificial intelligence (AI) in healthcare, particularly radiology, requires its unbiased and fair development and implementation, starting with the constitution of the scientific community.

**Purpose:**

To examine the gender and country distribution among academic editors in leading computer science and AI journals.

**Material and Methods:**

This cross-sectional study analyzed the gender and country distribution among editors-in-chief, senior, and associate editors in all 75 Q1 computer science and AI journals in the Clarivate Journal Citations Report and SCImago Journal Ranking 2022. Gender was determined using an open-source algorithm (Gender Guesser™), selecting the gender with the highest calibrated probability.

**Result:**

Among 4,948 editorial board members, women were underrepresented in all positions (editors-in-chief/senior editors/associate editors: 14%/18%/17%). The proportion of women correlated positively with the SCImago Journal Rank indicator (ρ = 0.329; *p* = .004). The U.S., the U.K., and China comprised 50% of editors, while Australia, Finland, Estonia, Denmark, the Netherlands, the U.K., Switzerland, and Slovenia had the highest women editor representation per million women population.

**Conclusion:**

Our results highlight gender and geographic disparities on leading computer science and AI journal editorial boards, with women being underrepresented in all positions and a disproportional relationship between the Global North and South.

## Introduction

The development, use, and popularity of artificial intelligence (AI) applications in radiology have increased rapidly in recent years.^1^ As of 2022, the U.S. Food and Drug Administration had approved a total of over 500 AI- and machine learning-enabled medical devices, of which more than 75% (*N* = 391) are dedicated to the radiological discipline.^2^ Among various use cases, these AI-powered tools can assist in medical image analysis reaching expert-level results, the transformation of free-text into structured radiology reports, image reconstruction, or radiation dose optimization.^3–6^ Since a significant share of medical AI devices is related to radiology, scientific AI, and informatics journals publishing medical AI content can act as important intermediaries between AI developers, scientists, and radiologists, presenting, critically discussing, and disseminating the latest applications across the professional community.^2,7^ However, while there are debates about bias, fairness, and diversity of radiological AI applications, there is currently a lack of research on the diversity of experts and scientists who develop new AI products and strategies, which extends to the academic publishing of AI literature.^8–10^

Despite the fundamental role of (external) peer review in academic publishing to achieve an unbiased and fair expert evaluation of the studies conducted, editors of scientific journals hold one of the most powerful positions in academic research, as they evaluate the suitability of manuscripts in the first instance and have the final decision-making authority in the publication process.^11–13^ Moreover, editorial board members guide and advise on publishing activities, directions, and processes and promote journals and research among their peers.^14^ Hence, the diversity of editorial board members is instrumental in making unbiased judgments, informing policies and best practices, and ultimately empowering diverse research, perspectives, and stakeholders.^15,16^ In the context of medical AI journals, diverse editorial boards may foster the development and publication of more accurate and generalizable AI models that meet the needs of diverse patient populations, consequently enhancing diagnostic and treatment capabilities.^8,9,17^

Although previous studies have examined gender and geographical representations of editorial board members or authorships in radiology and other disciplines affected by medical AI, research on the diversity of academic AI journals remains limited.^10,18,19^ To promote diversity, address biases, and foster a more inclusive research community in the field of (medical) AI development and research, this study primarily aims to provide a first-time, up-to-date assessment of gender disparities and secondarily examines the geographical distribution among academic editors on the editorial boards of leading AI and computer science journals.

## Material and methods

This cross-sectional study was conducted in accordance with the STROBE guidelines. Because this study was based on publicly available data, institutional review board approval was not required following our institution’s policies.

### Data collection

We examined the gender and country distribution among editors-in-chief, senior, and associate editors of all Q1 journals in the category “Computer Science, Artificial Intelligence” of the Clarivate Journal Citations Report 2022 (*N* = 36) and each additional Q1 “Computer Science” journal in the top 50 ranks of the SCImago Journal Ranking 2022 (*N* = 39).^20,21^

Gender and affiliation country of the academic editors were collected by three readers (FB, CR, LCA; *N* = 25 journals each). Gender was inferred by analyzing each editor’s full name and affiliation country with the open-source algorithm Gender Guesser*™*, selecting the gender with the highest calibrated probability.^22^

### Statistical analysis

Statistical analysis was performed using SPSS (version 28.0.1.0) and R (version 4.2.1) with the “tidyverse” and “sf” packages.^23^ The “naturalearth” package and Wikipedia’s list of countries by sex ratio were used for mapping affiliation countries.^24^ Median and range were reported for the proportion of women academic editors, impact factor (IF), journal citation indicator (JCI), total citations, and SCImago journal rank (SJR) indicator. Normal distribution was tested using the Kolmogorov–Smirnov test. Spearman's ρ was used to determine the correlation between the share of women academic editors and the aforementioned research impact measures. The Mann–Whitney U test was used to compare the impact measures and the proportion of women senior and associate editors between journals with only men and at least one woman editor-in-chief. Journals with missing data were excluded from sub-analyses. A two-sided *p* value <.05 was considered significant.

## Results

### Journal impact measures and origin

The SJR indicator was available for 73 of 75 journals (median: 3.84, range: 1.37–13.21). IF was available for 72 journals (median: 9.93, range: 3.92–29.23). Total citations number (median: 14,132, range: 434–174,343) and JCI (median: 2.15, range: 0.73–6.36) were available for 74 journals each. Most journals were registered in the U.S. (*N* = 43), followed by the U.K. (*N* = 15), Netherlands (*N* = 13), Switzerland (*N* = 2), Singapore (*N* = 1), and Germany (*N* = 1).

### Gender diversity of editorial boards and association with impact measures

Table 1 displays the distribution of editorial board positions by gender and country. We collected data on gender for 4,948 academic editors, of whom 4,083 (83%) were classified as men and 865 (17%) as women. The median overall women share was 18% (range: 0%–60%). Overall, women editors were underrepresented in editor-in-chief (*N* =12/85 (14%); median: 0%, range: 0%–100%), senior (*N* = 85/460 (18%); median: 11%, range: 0%–100%), and associate editorial board positions (*N* = 768/4,403 (17%); median: 18%, range: 0%–67%). In total, 65 journals had one, and ten journals had two editor-in-chief positions. Seven of the twelve women editors-in-chief held the position alone, while five co-chaired the position with a man editor-in-chief.

**Table 1.** Gender distribution of editors-in-chief, senior editors, associate editors, and overall by country.

Variable	Editors-in-chief		Senior editors		Associate editors		Overall	
	Men	Women	Men	Women	Men	Women	Men	Women
**Total number (%)**	73 (86)	12 (14)	375 (82)	85 (18)	3,635 (83)	768 (17)	4,084 (83)	865 (17)
**Number by country (%)**								
United States	23 (85)	4 (15)	137 (78)	39 (22)	924 (78)	266 (22)	1,084 (78)	309 (22)
China	2 (67)	1 (33)	24 (89)	3 (11)	607 (85)	103 (15)	633 (86)	107 (14)
United Kingdom	7 (100)	0	32 (91)	3 (9)	245 (77)	72 (23)	284 (79)	75 (21)
Canada	2 (67)	1 (33)	16 (76)	5 (24)	142 (82)	31 (18)	160 (81)	37 (19)
Italy	3 (100)	0	15 (71)	6 (29)	172 (82)	39 (18)	190 (81)	45 (19)
Australia	7 (78)	2 (22)	14 (74)	5 (26)	140 (87.5)	20 (12.5)	161 (86)	27 (14)
France	3 (60)	2 (40)	17 (89)	2 (11)	128 (84)	25 (16)	148 (84)	29 (16)
Germany	5 (100)	0	9 (69)	4 (31)	112 (84)	21 (16)	126 (83)	25 (17)
Spain	3 (100)	0	7 (100)	0	108 (84)	21 (16)	118 (85)	21 (15)
Hong Kong	2 (100)	0	9 (82)	2 (18)	83 (86)	13 (14)	94 (86)	15 (14)
Taiwan	3 (75)	1 (25)	3 (75)	1 (25)	69 (95)	4 (5)	75 (93)	6 (7)
Singapore	3 (100)	0	8 (80)	2 (20)	77 (91)	8 (9)	88 (90)	10 (10)
Japan	2 (100)	0	7 (100)	0	94 (91)	9 (9)	103 (92)	9 (8)
Netherlands	0	0	6 (100)	0	61 (78)	17 (22)	67 (80)	17 (20)
India	1 (100)	0	7 (78)	2 (22)	84 (88)	11 (12)	92 (88)	13 (12)
Sweden	1 (100)	0	7 (100)	0	34 (83)	7 (17)	42 (86)	7 (14)
Switzerland	0	0	6 (100)	0	31 (77.5)	9 (22.5)	37 (80)	9 (20)
South Korea	1 (100)	0	3 (100)	0	44 (90)	5 (10)	48 (91)	5 (9)
Brazil	0	0	2 (67)	1 (33)	41 (72)	16 (28)	43 (72)	17 (28)
Belgium	0	0	5 (83)	1 (17)	27 (84)	5 (16)	32 (84)	6 (16)
Denmark	1 (100)	0	1 (25)	3 (75)	16 (80)	4 (20)	18 (72)	7 (28)
Norway	0	0	2 (100)	0	17 (81)	4 (19)	19 (83)	4 (17)
Israel	0	0	5 (100)	0	20 (83)	4 (17)	25 (86)	4 (14)
Finland	1 (100)	0	4 (80)	1 (20)	20 (77)	6 (23)	25 (78)	7 (22)
Austria	1 (100)	0	0	0	25 (96)	1 (4)	26 (96)	1 (4)
Greece	0	0	1 (100)	0	23 (92)	2 (8)	24 (92)	2 (8)
Portugal	0	0	3 (100)	0	25 (78)	7 (22)	28 (80)	7 (20)
Ireland	1 (50)	1 (50)	3 (100)	0	7 (78)	2 (22)	11 (79)	3 (21)
Poland	0	0	1 (100)	0	32 (97)	1 (3)	33 (97)	1 (3)
Mexico	0	0	0	0	28 (87.5)	4 (12.5)	28 (87.5)	4 (12.5)
Turkey	0	0	1 (50)	1 (50)	29 (85)	5 (15)	30 (83)	6 (17)
Chile	0	0	2 (100)	0	14 (87.5)	2 (12.5)	16 (89)	2 (11)
Qatar	0	0	2 (100)	0	15 (100)	0	17 (100)	0
Czech Republic	0	0	1 (100)	0	9 (69)	4 (31)	10 (71)	4 (29)
South Africa	0	0	1 (100)	0	11 (79)	3 (21)	12 (80)	3 (20)
Iran	0	0	2 (100)	0	8 (100)	0	10 (100)	0
Saudi Arabia	0	0	1 (100)	0	10 (91)	1 (9)	11 (92)	1 (8)
United Arab Emirates	0	0	5 (100)	0	2 (100)	0	7 (100)	0
Cyprus	0	0	0	0	6 (100)	0	6 (100)	0
Romania	0	0	0	0	9 (90)	1(10)	9 (90)	1 (10)
Russia	0	0	0	0	4 (100)	0	4 (100)	0
Egypt	0	0	0	0	6 (100)	0	6 (100)	0
Hungary	0	0	0	0	5 (100)	0	5 (100)	0
Lebanon	0	0	0	0	3 (100)	0	3 (100)	0
Malaysia	0	0	0	0	1 (17)	5 (83)	1(17)	5 (83)
Slovenia	0	0	1 (100)	0	17 (85)	3 (15)	18 (86)	3 (14)
Thailand	0	0	0	2 (100)	1 (50)	1 (50)	1(25)	3 (75)
Iceland	0	0	1 (100)	0	1 (100)	0	2 (100)	0
Bangladesh	0	0	0	0	2 (100)	0	2 (100)	0
Colombia	0	0	0	0	2 (100)	0	2 (100)	0
Estonia	0	0	0	0	2 (50)	2 (50)	2 (50)	2 (50)
Liechtenstein	0	0	0	0	2 (100)	0	2 (100)	0
Luxembourg	0	0	0	0	4 (100)	0	4 (100)	0
Pakistan	0	0	0	0	3 (100)	0	3 (100)	0
Argentina	0	0	1 (100)	0	1 (33)	2 (67)	2 (50)	2 (50)
Peru	0	0	0	0	1 (100)	0	1 (100)	0
Philippines	0	0	0	1 (100)	0	0	0	1 (100)
Sri Lanka	0	0	0	1 (100)	0	0	0	1 (100)
Tunisia	0	0	1 (100)	0	3 (100)	0	4 (100)	0
Ukraine	0	0	1 (100)	0	0	0	1 (100)	0
Guyana	0	0	0	0	1 (100)	0	1 (100)	0
Malta	0	0	0	0	1 (100)	0	1 (100)	0
Cuba	0	0	0	0	1 (100)	0	1 (100)	0
Azerbaijan	0	0	0	0	1 (100)	0	1 (100)	0
Jordan	0	0	0	0	1 (100)	0	1 (100)	0
Kuwait	0	0	0	0	1 (100)	0	1 (100)	0
Lithuania	0	0	0	0	1 (50)	1 (50)	1 (50)	1 (50)
Serbia	0	0	0	0	2 (100)	0	2 (100)	0
Macao	0	0	0	0	3 (100)	0	3 (100)	0
Slovakia	0	0	0	0	1 (100)	0	1 (100)	0
Uruguay	0	0	0	0	1 (100)	0	1 (100)	0
Vietnam	0	0	0	0	0	1 (100)	0	1 (100)
Not applicable	1 (100)	0	1 (100)	0	15 (100)	0	17 (100)	0

**Fig 1.**
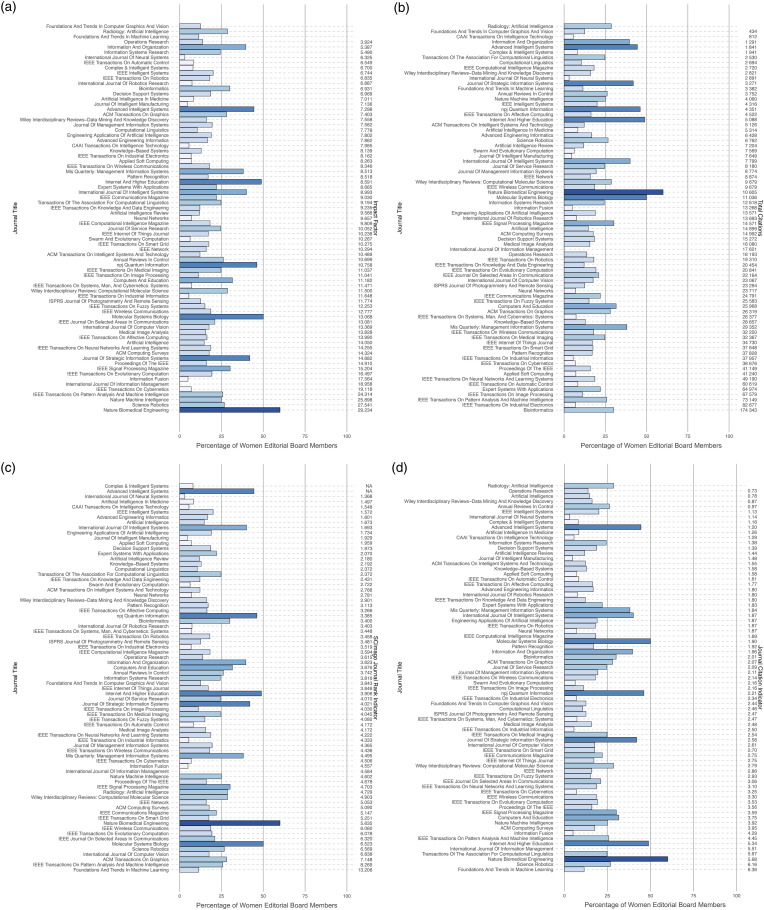
Percentage of women editorial board members among all computer science journals analyzed in relation to the corresponding impact factor (a), total citations (b), SCImago journal rank indicator (c), and journal citation indicator (d). Notes: (a) Radiology: Artificial Intelligence, Foundations, and Trends in Machine Learning, and Foundations and Trends in Computer Graphics and Vision did not receive an impact factor yet. b, (d) Radiology: Artificial Intelligence is not listed in the Journal Citation Report 2022. (c) Complex & Intelligent Systems and Advanced Intelligent Systems are not listed in the SCImago journal ranking.

**Fig 2.**
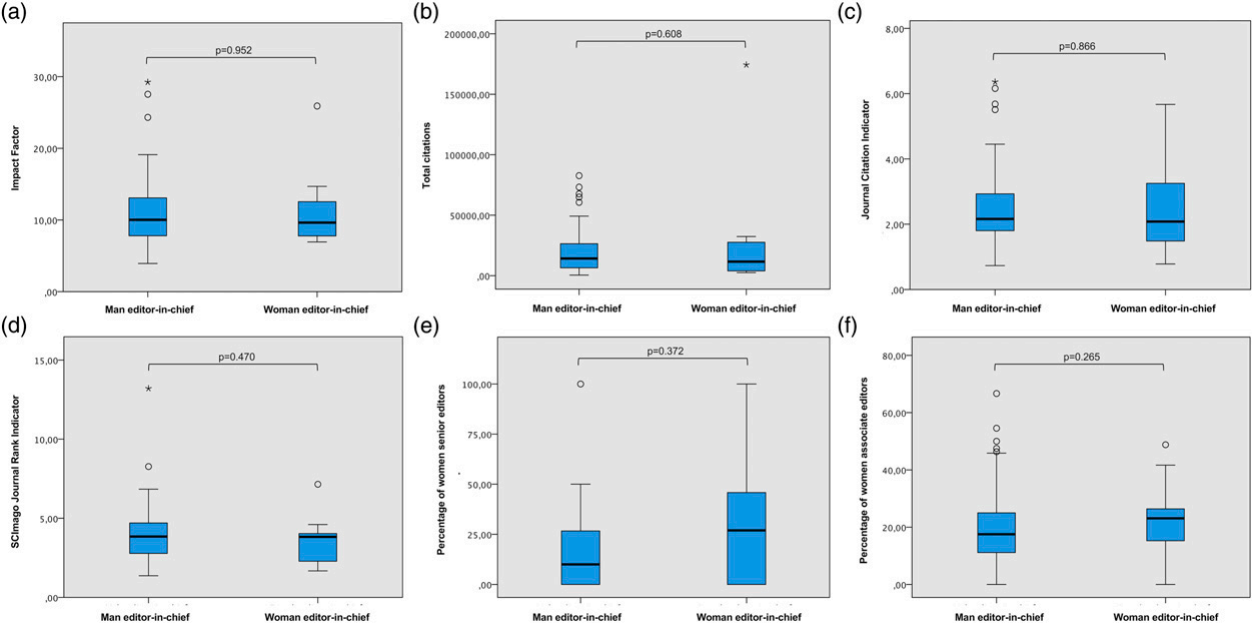
Boxplots show the distribution of values between computer science journals with only male editors-in-chief and at least one woman editor-in-chief for impact factor (a), total citations (b), journal citation indicator (c), SCImago journal rank indicator (d), percentage of women in senior editorial board positions (e), and percentage of women associate editors (f). Notes: *p* values display the results of the Mann–Whitney U test, which was used because of the nonparametric distribution of the data. There were no significant differences for all variables.

Fig. 1 illustrates all journals analyzed and their proportion of women editors compared to their respective IF, JCI, total citations, and SJR indicator. Out of 75 journals examined, 73 (97%) had editorial boards with a higher proportion of men, with one journal having no women on the editorial board. Of the remaining two journals, one featured an equal number of men and women academic editors, and one had a women-dominated editorial board. The women's share of editorial boards did not significantly correlate with IF (ρ = 0.123; *p* = .304), JCI (ρ = 0.218; *p* = .062), and total citation number (ρ = –0.104; *p* = .379), but positively correlated with the SJR indicator (ρ = 0.329; *p* = .004). IF, JCI, SJR indicator, total citation number, and the percentage of women editors in senior/associate editorial board positions did not significantly differ between journals with only men editors-in-chief and journals with at least one woman editor-in-chief (see Fig. 2).

### Country composition of editorial boards

Please refer to Table 1 to view the complete list of editors and editorial board positions per country. Overall, 72 different countries were identified. Most editors, regardless of their editorial board position, were affiliated with U.S. institutions (editors-in-chief: *N* = 27/84 (32%), senior editors: *N*= 176/459 (38%), and associate editors: *N* = 1,190/4,388 (27%)), followed by Australia for editors-in-chief (*N* = 9 (11%)), the U.K. for senior editors (*N* = 35 (8%)), and China for associate editorial board positions (*N* = 710 (16%)).

**Fig 3.**
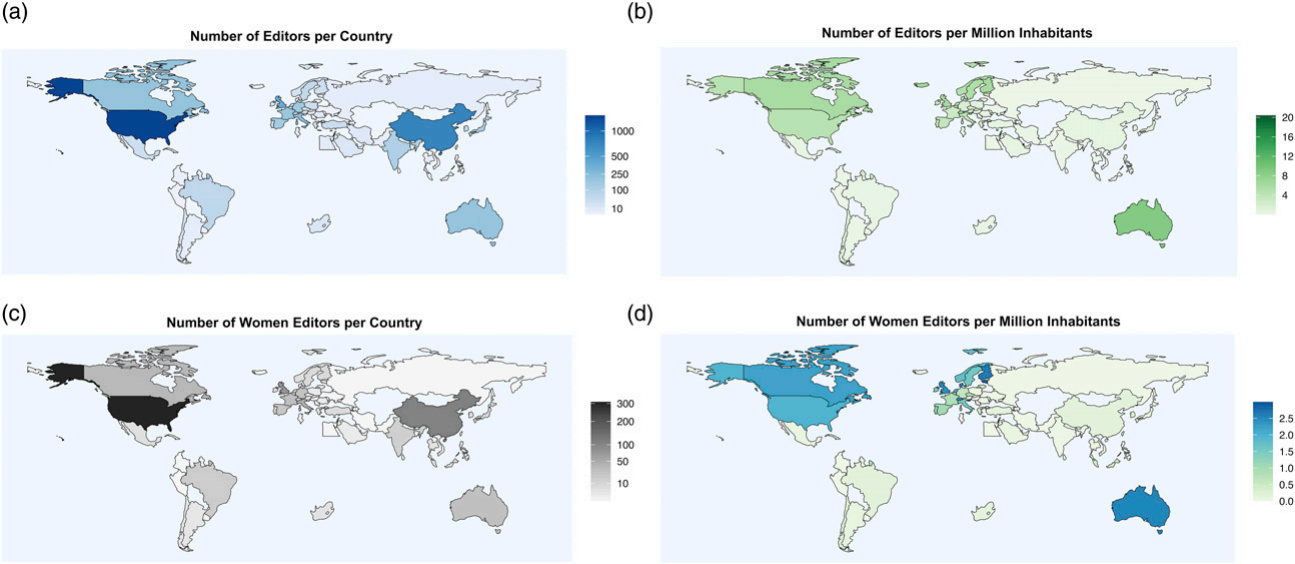
World maps display the absolute distribution of computer science journal editors per country (a, blue), number of editors per million inhabitants (b, green), number of women editors per country (c, grey/black), and number of women editors per million women (d, green/blue). Notes: While in absolute numbers, most editors (a) and women editors (c) are based in the U.S. and China, the number of editors per million inhabitants (b) shows that in relation to the population, Australia, Canada, the U.K., the northern and southern European countries alongside with the U.S. dominate, but no longer China. In contrast, the number of women editors per million women population (d) displays Australia, Finland, Estonia, Denmark, the Netherlands, the U.K., Switzerland, and Slovenia as the leading countries.

Fig. 3 depicts world maps representing the geographical distribution of academic editors in our study. When analyzing the geographical distribution of all editors or women editors in absolute terms, a pronounced representation from the U.S. and China was observed. However, this changes if the absolute number is normalized to editors per million inhabitants, where Canada, Australia, the U.K., and northern and southern European countries were predominantly represented alongside the U.S. but no longer China. When evaluating the number of women editors per million women population, Australia, Finland, Estonia, Denmark, the Netherlands, the U.K., Switzerland, and Slovenia accounted for the highest ratio of women.

## Discussion

In the present study, women editorial board members were underrepresented in 73 of 75 computer science and AI journals, with a particularly pronounced disparity in the position of editor-in-chief. The proportion of women on editorial boards showed a positive but weak correlation with the SJR indicator, while there was no significant correlation with IF, JCI, and total citations. Most editors were based in the U.S. and China, followed by Canada, Australia, the U.K., and northern and southern European countries, indicating a disproportionate relationship between the Global South and North and the most populous countries and residence of academic editors.

The underrepresentation of women on editorial boards extends beyond the fields of computer science and AI and is also evident in radiological research and other academic disciplines.^18,19,25–27^ Focusing on diversity research of radiology journal editorial boards, a 2018 study by Abdellatif et al. revealed that out of 460 editorial board members, only 88 (19%) were women, which is consistent with the overall women share of 17% (865 out of 4,948) in our study.^18^ In a recent 2022 study by Joshi et al., the representation of women on radiological editorial boards was even lower, with 2.4% (1 out of 42) for editors-in-chief, 17.8% (24 out of 135) for deputy editors, and 13.6% (345 out of 2,545) for all editorial board members.^25^ On the other hand, Alkhawtani et al. showed that out of 57 radiological journals in 2021, only 5 (8.8%) had a woman as the sole editor-in-chief, while of 4,176 editorial board members, the median proportion of women was 21.5%, indicating a varying distribution of women depending on the investigated radiological journals and editorial positions.^26^ Still, all studies display a significant lack of women in all editorial board positions of radiology journals. Given the exponential growth of medical AI-related content not only in computer science but also in academic radiology in recent years, this could likewise hinder the diversity of medical AI research, for example, by discouraging a variety of stakeholders from submitting their contributions, fostering an environment where dominant groups overshadow others, and limiting the range of research topics or inadvertently introducing biases in algorithms.^28–31^ In medicine, this carries the risk of disparities in medical diagnostic, therapeutic, and prognostic outcomes for marginalized groups.^8^ However, in this respect, not only a diverse editorial board but also the diversity of peer review and double-blinding of manuscripts play a fundamental role.^32^ Moreover, especially in the field of medical AI, it is essential to emphasize the importance of diversity, equity, and equality in order to comply with the fundamental principles of biomedical ethics.^33^

Finally, six journals stand out in our study for having a comparatively higher proportion of women editorial board members, represented by dark blue bars in Fig. 1, namely, Nature Biomedical Engineering (the only journal with more than 50% women editorial board members), npj Quantum Information, Molecular Systems Biology (all part of Nature portfolio), Internet and Higher Education, Journal Of Strategic Information Systems (both Elsevier), and Advanced Intelligent Systems (published by Wiley). While all three publishers have made public commitments to diversity in the past, they host 27 other journals with significantly lower percentages of women editorial board members in our study.^34–36^ This discrepancy may suggest that the heightened presence of women in these journals is coincidental rather than a deliberate result of these commitments, underscoring the need for sustained efforts to consistently ensure diversity in the field of (medical) AI research.

In terms of geographical representations of academic editors, we could identify a disproportionate relationship between the most populous countries and where academic editors in computer science and AI journals are based. Despite accounting for 35% of the world population, China and India account for only 17% and 2% of editors, respectively, while 28% of editors are affiliated with the U.S., which accounts for only 4% of the world population.^37^ It is noteworthy that India and the countries ranked fourth through tenth (Indonesia, Pakistan, Nigeria, Brazil, Bangladesh, Russia, and Mexico) in the 2022 world population ranking are not even among the ten countries with the most editors in our study. Moreover, this reveals an underrepresentation of editors from countries in the Global South. Possible explanations include the interplay between most computer science journals registered in the U.S. and Europe, popular university locations, and the migration of researchers from the Global South to the North.^38,39^ In addition, researchers in the Global South are more likely to face challenges such as limited research funding, lack of infrastructure and training, and restricted access to academic networks compared to their peers from the Global North.^38–40^ This inequity is even more concerning when contemplating the deployment of AI in healthcare within underrepresented regions. For example, countries in the Global South, which often face a dearth of medical professionals, could immensely benefit from AI-supported diagnostics, such as deep-learning algorithms for segmenting and classifying chest X-rays.^41,42^ Yet, the usefulness of these tools is contingent on their accuracy and representativeness.^43^ If the training data for medical AI tools is predominantly sourced from the Global North, there is a risk that algorithms are performing poorer in recognizing certain conditions inherent to other patient demographics.^42,44,45^ This lack of inclusivity and potential bias in AI may hamper its applicability and success in varied global settings.

Limitations of our study include potential misclassification of gender using Gender Guesser™ due to varying associations between names and gender across cultural, regional, and historical contexts.^46^ In addition, it is essential to note that Gender Guesser™ is designed to predict only binary gender, that is, categorizing individuals as either male or female. This inherently excludes individuals who do not strictly identify as such, including those who identify as non-binary, genderqueer, or other dimensions in the gender spectrum. Lastly, our cross-sectional analysis represents a snapshot and does not capture changes over time.

In conclusion, this paper highlights the underrepresentation of women academic editors in leading computer science and AI journals and emphasizes the unequal representation of editors from the Global South. Future research should further increase gender sensitivity, track progress, and help address gender and geographical imbalances to promote ethical, inclusive medical AI that reflects the diverse patient population it serves, aiming to reduce bias in medical diagnosis and treatment and empowering generalizable AI.

## Data Availability

The collected and analyzed dataset is openly available at: https://figshare.com/s/b75bd403805b9ec0e9b0 [non-permanent anonymized link until publication].
